# Supplementary material for: Patient health records and whole viral genomes from an early SARS-CoV-2 outbreak in a Quebec hospital reveal features associated with favorable outcomes
Source: PLoS One. 2021 Dec 2;16(12):e0260714. doi: 10.1371/journal.pone.0260714 (PMC8638998; doi:10.1371/journal.pone.0260714)
Supplement: Attachment — Submitted filename: Response to Reviewers 2.docx [file pone.0260714.s016.docx]

**Comments to the Author**

**Reviewer #1: Authors have addressed all comments appropriately. Thank you for the opportunity to review your work. Congratulation!**

Thank you for your input.

**Reviewer #2: Review of the paper "Patient health records and whole viral genomes from an early SARS-CoV-2 outbreak in a Quebec hospital reveal features associated with favorable outcomes"**

**General Comments:**

**The authors exerted good effort to address my comments. There are few points that still need to be improved. The authors have chosen to focus more in the revised version on the methodology and clinical associations. This is in my view good choice especially the sequencing data of the paper represents older wave of the viral evolution.**

**Specific Comments:**

**- The authors need to comment on the limitations of the sequencing that some positions were not covered by the genome and the authors guessed them using dominant VAF? How many of these variant existed and what is the number of the genomes affected by this?? How this would affect the results if there were wrong predictions.??**

The ARTIC Bioinformatics Standard Operating Procedure for nanopore requires that at least 20 reads per strand per amplicon are present to generate a consensus – we stuck to these parameters for consistency with other ARTIC-nanopore consensus sequences in GISAID. Regions not covered by at least 20 reads on either strand are therefore not included in the consensus (NNNNN...). *At no point do we ‘guess’ a variant.* The reviewer may be referring to situations when an allele frequency below ~0.9 is observed, which causes the default variant calling pipeline to occasionally emit ambiguous “N” variants instead. In these situations, which are independent of coverage, we extracted the major allele in the consensus sequence, which is default behaviour for haploid genomes. This post-processing we performed corrects a recurring artifact in the ARTIC pipeline when using medaka, which has been reported by ARTIC pipeline developers (<https://community.artic.network/t/medaka-longshot-pipeline/107>).

**- The authors need to mention their effort in inspecting the S and N genes and how the missing positions do not affect much their conclusion as they mentioned in the response to my revision. I missed this part in the results section.**

Thank you for this recommendation. We added the summary of this analysis (c.f. previous response to reviewers) on page 8 and 10-11 of the results, Specifically:

*Of note, 42/237 genomes with ≥80% completeness had an incomplete S gene and 90/237 had an incomplete N gene. The latter harbors one of the consistently less abundant amplicons from the ARTIC V3 PCR amplification scheme (S1 Fig). However, only 4 unique mutations (7 mutations in total) were observed in 147 genomes with complete N genes, suggesting that the missing sequences are unlikely to contain many mutations. Few mutations were also observed for the S gene; besides the D614G mutation (present in all but one genome), 9 genomes had an A24782G mutation (N1074D substitution) and 7 had a G21641T (A27S substitution).*

*…*

*All 9 A24782G mutations in the N gene (see above) were uniquely present in haplogroup IIa genomes, whereas 5/7 G21641T mutations in the S gene were present in haplogroup IIb genomes (the remaining 2 in haplogroup III).*

**- For best practice, the user should mention the sequencing parameters (depth and coverage) in their dataset and compare this to the usually obtained results as per the paper of Bull et al paper. Bull et al. mentioned coverage of 99.6% and tested sensitivity down to 50X read depth: They stated that sensitivity and precision of variant detection were strongly influenced by sequencing coverage, showing a sharp decline below ~50-fold coverage depth. (This can mean that one can target 99% coverage at 50X depth.) The authors need to comment on that and put the reader in context about the sequencing quality in this paper. How many of their sequences reached that level and why they still retained them in the analysis.**

The depth and coverage from our dataset are illustrated in Table 1 as well as detailed in Supplementary Figure 1. As for comparing the depth and coverage we obtained to the ones presented by Bull *et al.*, it is important to note that *their methodology is distinct and was developed after we published the consensus genomes* associated with this publication. Their protocol is optimised for nanopore sequencing (~2.5 kb-long amplicons), whereas the ARTIC V4 primer scheme we used was intended for compatibility across Illumina and Nanopore platforms, for broader community adoption.

Furthermore, the Bull et al. study and most SARS-CoV-2 sequencing protocols typically one process samples with higher viral mRNA quantities (Ct scores < ~30 or CN scores < ~20, depending on local recommendations). As we shown in Figure 1B, lower scores (more RNA) are associated with more even amplicon coverage and, consequently, higher genome completeness. As mentioned in the discussion, *a unique feature of our study is that we included samples with lower RNA abundance* (i.e. leading to low coverage), which complicates variant calling and lineage assignment, but can provide information on intra-host variation and viral evolutionary dynamics–two aspects not typically addressed in studies tacking genomic epidemiology alone.

We would like to remind the reviewer that a minimum of 40-fold coverage (20 on both strands of an amplicon) are required to produce a consensus sequence with the ARTIC bioinformatics SOP. Given the dynamics of RT-PCR and the ARTIC V3 primer scheme, some amplicons have systematically higher or lower coverage than others (see comments in the previous response), which can cause coverage dropouts as illustrated in Supp Figure 1. Nevertheless, we obtained a median completeness of 97.7% with short amplicons (about 400 bp) for all genomes, with CN ranges between 0-31. This includes 70 genomes with full-coverage (99.6%), or 26.5% of our samples (*a phrase was added to specify this in the results on page 6*). In contrast, Bull et al. obtained 99.6%, or the maximum possible with ARTIC primer schemes, using longer amplicons and Ct ranges ≤ 29. N.B. a Ct score of 33 is approximately equivalent to a CN score of 22.

We believe that readers interested in the sequencing quality of our study will appreciate the detailed metrics provided in Figure 1, Supplementary Table 1, Supplementary Figure 1 and the summary in the body of the Results section, as well as recognizing the use of the internationally-implemented ARTIC standard sequencing and bioinformatics protocol used by hundreds of laboratories sequencing SARS-CoV-2. Therefore, we do not think this should be further emphasized.

**- The authors need to state that their method worked well compared to Pangolin/Nextstrain only for this specific data set, and this cannot be generalized to other data-sets due to the lack of more extensive experimentation using larger sequencing set and more lineages.**

Recently, Mostefai *et al.*, [(Mostefai et al. 2021)](https://paperpile.com/c/jk4kzE/OjIN) also showed that this method can be used on larger sequencing sets (i.e. all 2020 SARS-CoV-2 genomes in GISAID), as well as more lineages. Thus, we believe this method can be used on datasets other than the one presented in this paper.

**- I would drop marketing statements like price of nanopore (CA$50 per sample); or if it is crucial for some reason to mention that, then plz state the cost of other technologies as well, taking sequencing parameters (sequencing and depth) per sample to reach accepted level of accuracy into consideration.**

We agree with the reviewer and the statements were removed in the updated manuscript.
